# Trick or treat: Social Media’s dissemination power of ophthalmologic information in the pandemic context

**DOI:** 10.22336/rjo.2021.26

**Published:** 2021

**Authors:** Consuela-Mădălina Gheorghe, Victor Lorin Purcărea, Iuliana-Raluca Gheorghe

**Affiliations:** * Department of Marketing and Medical Technology, “Carol Davila” University of Medicine and Pharmacy, Bucharest, Romania

**Keywords:** teleophthalmology, teleconsultation, Social Media group, patient satisfaction, health care consumer satisfaction, physician satisfaction

## Abstract

Social Media in the COVID-19 pandemic context has become a real dissemination medium of ophthalmology information for both physicians and health care consumers. This trend of sharing information has revealed new and innovative interventions in Ophthalmology such as teleophthalmology on Social Media by providing synchronous and asynchronous consultations, education, and prevention solutions as well as scientific research findings. This paper is a review of the current challenges and limitations faced by ophthalmologists and health care consumers during the COVID-19 pandemic.

## Introduction

For more than a year already, the world has been facing a major healthcare crisis triggered by the outbreak of a new coronavirus induced infectious respiratory disease, known as COVID-19 [**1**]. In the pandemic context, both essential and non-essential healthcare services have been forced to be provided on a limited basis in most high-risk countries in order to limit the spread of the disease. However, such drastic measures have not induced a collapsed stage in the evolution of the healthcare systems around the world, as countries had to adapt and to implement technology-driven medical consultations [**2**].

Along with the development of several digital technologies that address clinical diagnoses and treatments for different diseases, the pandemic context was the harbinger of an emergent phase in medicine, shaped by the increased usage of telemedicine and teleconsultations, gaining more popularity in the COVID-19 era [**3**]. Telemedicine relies on the telecommunication technology and provides remote medical assistance by telephone, internet, or other networks [**4**].

During the COVID-19 pandemic, the constant request for ophthalmology services made most specialists adapt and use teleophthalmology shared on Social Media groups for the diagnosis and treatment of specific eye diseases, as well as for research, and education [**5**]. Video-consultations have been introduced in the ophthalmology practice as an alternative to the conventional consultation in order to reduce the risk of getting or spreading the COVID-19 infection that may be associated with the close contact between the health care consumers and the ophthalmologists during a slit-lamp examination or between health care consumers in the waiting rooms. Although this measure has significantly contributed to the achievement of high levels of infection prevention, and at the same time ensured proper eye care for health care consumers, the potential power of Social Media has been evaluated, and showed a remarkable success not only in usefulness but also in terms of change in clinical and academic practice [**6**].

From a health care perspective, Social Media platforms such as Twitter and Facebook are increasingly used to seek health-care information, as online medical groups continue to develop, the number of health care consumers interested in seeking help online and getting empowered grows [**7**,**8**]. The Social Media groups are used by individuals to share stories, exchange information, or engage in discussions with other users or peers in the form of written or video messages and comments. Furthermore, Social Media has been largely adopted in medical education [**9**] because many dedicated online communities and groups have been built around prevention and interaction with the aim of health care consumers to assess and to find information about their diseases and to express their questions and fears regarding their conditions, as well as improve their well-being and promote social integration [**6**].

From a physician perspective, Social Media offers the opportunity to establish an online international and national interaction, provides an easy method to present a clinical and academic or scientific experience to other peers, and, at the same time, promotes the communication between academic peers and between physicians and the general population, implicitly, the health care consumers. Starr et al. (2020) highlighted that 17.5% of the private health care organizations and 15% of the public health care organizations were offering telemedicine services before the pandemic, even if the impact of this type of consultations on the outcomes are still unknown [**10**]. In the ophthalmology practice, most specialists stated that they have low confidence in the use of telemedicine for the eye care prior to COVID-19 [**11**]. Due to the pressure felt by the pandemic, most ophthalmologists adopted the teleconsultations and shared information on the Social Media. However, there is still limited information about the consequences of using Social Media on both health care consumers and specialists. Thus, the aim of this paper was to uncover the dissemination power of Social Media for both health care consumers and ophthalmology specialists during the COVID-19 pandemic.

## Challenges of Social Media for ophthalmologists

The constant innovations in the technology and surgical techniques, as well as the emergent developments in the basic and clinical research have made Ophthalmology a field in which Social Media may bring significant benefits and play an important role in delivering core information. As such, Social Media has proved to be useful for the teaching process of Ophthalmology medical students, residents, and specialists by expanding their knowledge and providing up-to-date clinical data [**6**]. In addition, video materials play a fundamental role in the teaching process of ophthalmology specialists and resident surgeons due to their low costs and high accessibility [**12**]. Moreover, several ophthalmology conferences have been held online and tweets have been shared on Facebook and Instagram by using the recognized official hashtags of the conferences.

With the help of Social Media, teleophthalmology - the ophthalmology video-consultation - is preferred, as the physician may see the patients directly, look for general eye signs and establish a diagnosis, advise the patients on management care, counsel them in an interactive manner to increase the adherence to treatment, as well as clarify all the inquiries of the health care consumers [**13**].

As a practice to patient care orientation, teleophthalmology has successfully been used in the treatment and monitoring of a vast palette of ophthalmological diseases such as suspected glaucoma [**14**], cataract screening [**15**], diabetic retinopathy, age-related macular degeneration and retinopathy of prematurity, anterior segment imaging, telementoring and low vision consultation [**3**] and, in addition, it effectively reduced the number of unnecessary referrals to eye care organizations [**16**]. Further, teleophthalmology can successfully help in assisting more complex surgical procedures via telementoring, which can play an important role in maintaining qualitative standards of medical care under pressure circumstances [**17**].

To reach an efficient ophthalmology approach, a distinction between asynchronous (store-and-forward) and synchronous (live video consultation) teleophthalmology methods is required. Another method is a hybrid consultation, which combines previous device-based investigations with the live video consultation assistance. In most developed countries, teleophthalmology is based on the asynchronous model, meaning the store-and-forward mode. [**18**]. In store-and-forward mode, patients’ electronic medical records, laboratory results, slit lamp and fundus images, as well as the audio or video-clips (e.g., eye movements, pupillary examination) are performed by an optometrist and, consequently, forwarded to a specialist, who reviews the referral at a convenient time, whereas the real-time mode initiates interactive services, such as audio telephone calls or videoconferencing and remote monitoring methods. Most ophthalmologists prefer the real-time teleconsultation because it helps in the sorting of acute cases and in the management of referrals, reassurance, reevaluation of care plans, as well as rescheduling of upcoming appointments or surgical procedures. Since any virtual consultation avoids the physical contact and the crowding of health care organizations, it may also limit the spread of the coronavirus and ensure the safety of health care professionals and the health care consumers [**19**].

**Fig. 1:**
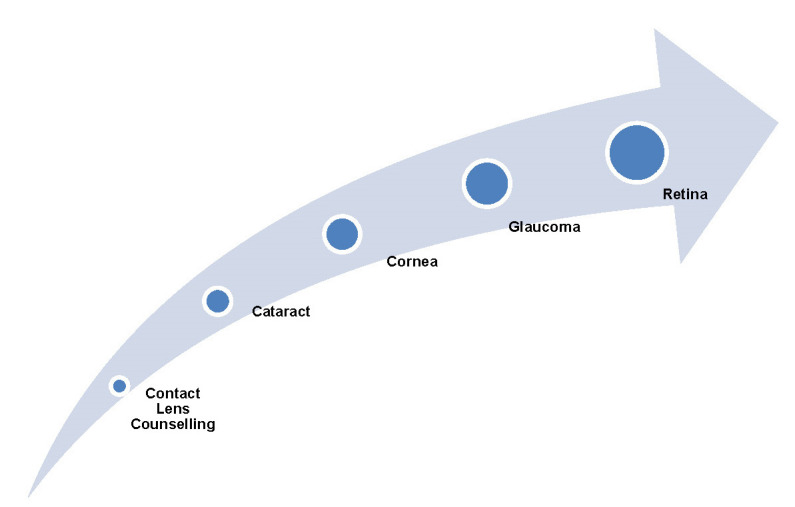
The eye diseases that proved to be successfully addressed in a teleophthalmology consultation

The eye diseases that proved to be successfully addressed in a teleophthalmology consultation are the following (**Fig. 1**) [**20**]:

• Cataract - Although it is an elective surgery, a delay in the diagnosis and treatment may convert the cataract case into an emergency procedure. Even if teleconsultation cannot do too much in the cataract case, it can be used as a tool to sort patients in determining the assessment of the vision status of an individual by occluding one eye and asking for finger counting at various distances or with the help of a relative.

• Cornea - Since the cornea can be visualized during a video consultation, it may help in establishing an accurate diagnosis by having a gross examination in various gazes.

• Glaucoma - The accurate diagnosis of glaucoma depends on the determination of the optic disc assessment and the visual field examination, known as perimetry. These can be easily performed during a teleconsultation, but the accurate intraocular pressure (IOP) measurement may be diagnosed with the help of a video teleconsultation if increased conjunctival congestion along with dry-eye symptoms are observed.

• Retina - Despite the limitations of not being able to perform a dilated examination on a teleconsultation, some patients with acute symptoms may still be assisted by a careful investigation of their medical history or symptomatology accordingly (**Table 1**).

**Tabel 1:** Retina symptomatology that can be addressed through teleconsultation

Symptom	Method	Outcome
Sudden drop in vision	Ask the patient to occlude the better eye and look at objects in the distance	Estimation of vision in the affected eye can be determined or vision acuity
Deterioration of vision	Gradual drop in vision methods	Age-related degeneration assessment
Trauma	Trivial injuries can be addressed through mobile applications, but severe blunt or penetrating trauma need multidisciplinary intervention	Referral to the closest emergency health care organization
Source: [**20**]		

Further, during the COVID-19 pandemic, teleophthalmology has also been used for second opinions in the shape of consultations and reassurance [**21**], and contact lens services counselling [**22**]. In this approach, certain professional ophthalmology associations have adopted Social Media to build communities and have many followers but still must grow [**23**]. According to Mahjoub et al. [**24**], the minority of self-identified users on Reddit, the Social Media platform, were ophthalmologists. This fact produces uncertainty, as anyone on the internet could assume the role of a “board-certified ophthalmologist” and, unfortunately disseminate inaccurate information. One potential solution to raise credibility is to ask interested ophthalmologists to self-identify and guide their patients towards useful resources that they have previewed.

## Challenges of Social Media for consumers of ophthalmology services

It is acknowledged that online communication platforms were widely used by ophthalmic patients before the pandemic and that the main utilities were for visual health education, communication between patients who share the same diseases, and for communication with the attending ophthalmologist [**25**]. Consequently, in the pandemic context, Social Media usefulness has been extended to all sub-specialties in ophthalmology along with the evolution of certain eye diseases and disorders. According to Mahjoub et al. [**24**], the ophthalmic patients’ posts on Reddit, the Social Media platform, were about seeking diagnoses (21%), surgical complications (11.5%), and second opinion medication options (6.50%). Moreover, the most frequently posted comments provided by other patients and optometrists concentrated on treatment advice (34.7%), advice for follow-up appointments with other ophthalmologists (15.4%), and sharing specific information (13.2%). Further, most ophthalmic patients’ emotions when posting Social Media comments were anxiety (13.4%), worry (12.2%), stress (7.32%), concern (7.32%), and fear (7.32%) [**24**,**26**].

According to Mansoor et al. [**3**], the most frequently requested service for teleophthalmology during lockdown was red-eye (16.70%), which was associated with seasonal ocular allergic disorders, followed by trauma (9.36%) and infectious keratitis-related consultations (4.33%). Similarly, while glaucoma is a chronic disease, and most patients require a lifelong therapy for its treatment, its care may be improved by finding and seeking information regarding the disease and treatment options, emotional support and sharing experiences [**27**], especially some advice regarding drug allergies or ocular surface problems [**3**]. Another study [**18**] investigated patient satisfaction with the Virtual Consultations that were used in the Ophthalmology practice during the COVID-19 pandemic and revealed that 95% of patients rated their overall experience with the virtual consultation as good and very good, and that all study participants will recommend the virtual consultation approach to others. However, middle-aged, and elderly patients with cataract, presbyopia, and stable posterior segment pathologies, namely, stable diabetic retinopathy and stable age-related macular degeneration, were more likely to wait for the face-to-face ophthalmology services rather than be evaluated through a teleconsultation [**3**]. 

Health care consumers with refractive surgery were the most highly active group on Social Media, because they are younger than most ophthalmic patients, and they search for information about treatment options and surgeons [**3**]. In addition, although there is an active participation of transplantation centres on Social Media, with the aim to increase social awareness on organ donation and transplantation among the general population; this has not contributed to the increase rates of corneal graft availability for corneal transplantation [**3**]. 

Although the online patient communities emphasize that “online advice can never replace an actual medical examination”, and that all posts should be moderated by self-identified ophthalmologists that may either approve comments to be posted or rejected, the largest presence on Social Media are the Patient advocacy groups that are most likely to address to the general public and not necessarily to ophthalmic professionals with tweets and posts about medical issues, prevention and education measures, educational events, fundraising opportunities, and recent advances in eye research [**23**]. On Social Media, there is a risk of spreading inaccurate advice and information that might inappropriately increase ophthalmic patients’ anxiety or even have greater consequences such as missing an important diagnosis [**28**,**29**]. In general, patient education topics on Social Media should be checked by board-certified ophthalmologists or optometrists and archived on the Social Media groups for potential health care consumers to access, as in the rapidly developing era of teleophthalmology, this online environment should become an ally to practicing clinicians.

## Current limitations of Social Media in Ophthalmology

The initiative of using Social Media to guide health care consumers and providers towards more accurate diagnoses or sharing treatment advice is exciting and new, especially in Ophthalmology. With all new technology advances and innovation practices, we should discuss the potential negative consequences of Social Media by investigating the perspectives of physicians, health care consumers and the quality of the provided information. As such, in considering the growth of Social Media in the COVID-19 context, there will surely be a push-back from providers, who believe it as encroaching on their field. Moreover, physicians, and, implicitly, ophthalmologists are at risk of getting an undesired mix of their private and personal lives within their professional and online profiles, raising serious ethical and potentially legal issues. They may become more exposed to abusive patients, who seek excessive contact with the attending ophthalmologist, whereas, health care consumers are at risk of data confidentiality loss, and, effective measures for health-related data protection are required. 

Further, online information on Social Media may be irrelevant, of poor quality and have low scientific accuracy, such as presenting exaggerated surgical outcomes and disregarding the risks associated with any surgical procedure, noted especially in cataract and refractive surgery, and retinal implant [**30**]. Thus, there is a rising need for medical professionals to advise patients online in a regulated manner, through an easy-to-use online patient education tool in the shape of a frequently asked questions section on the Social Media platform, where patients look for information that has previously been validated and endorsed by certified ophthalmologists.

Lastly, financial costs of teleophthalmology should be reported in terms of fixed and recurrent costs, and should be linked to the utilization of the services including projected management estimates, future growth in the ophthalmology field, the evolution and cost of technology involved and the desired return on investment. 

## Conclusion

During the COVID-19 pandemic, teleophthalmology served, in selected cases, as a complement to conventional consultations. However, continuous user-oriented technological development is crucial in this consultation process in order to become more widespread. 

The utopian challenge that a crisis becomes an opportunity is true in the pandemic context, as a fusion between the health sector and the optimal digital technology. In the digital and Social Media era, the power of these communication means is quickly implemented in the medical field, particularly in Ophthalmology, enabling fast and global dissemination of scientific information, improving scientific communication among specialists, and establishing favorable relationships between experts and health care consumers. The active online participation of health care professionals and patient online communities will be a major health care strategy to improve health education in the ophthalmic care, as teleophthalmology in Social Media is more likely to stay beyond the pandemic, as practices need to be adjusted and updated to the current telemedicine guidelines. 

**Conflict of Interest statement**

The authors state no conflict of interest.

**Acknowledgments**

None.

**Sources of Funding**

None.

**Disclosures**

None.
